# Supplementary material for: Single Cell Transcriptomic and Chromatin Profiles Suggest Layer Vb Is the Only Layer With Shared Excitatory Cell Types in the Medial and Lateral Entorhinal Cortex
Source: Front Neural Circuits. 2022 Jan 26;15:806154. doi: 10.3389/fncir.2021.806154 (PMC8826650; doi:10.3389/fncir.2021.806154)
Supplement: Supplementary file 14 [file Data_Sheet_1.ZIP › Deep_vs_Sup_Motifs/homerResults/motif1.similar.html]

motif1

## Information for motif1

C
G
A
T
C
A
T
G
A
T
G
C
A
G
T
C
C
G
T
A
C
T
G
A
C
T
A
G
G
C
T
A
G
C
A
T
G
C
A
T
  
Reverse Opposite:  

C
G
T
A
C
G
T
A
C
G
A
T
G
A
T
C
A
G
C
T
A
C
G
T
A
C
T
G
T
A
C
G
G
T
A
C
C
G
T
A
  

|  |  |
| --- | --- |
| p-value: | 1e-201 |
| log p-value: | -4.637e+02 |
| Information Content per bp: | 1.710 |
| Number of Target Sequences with motif | 4974.0 |
| Percentage of Target Sequences with motif | 53.50% |
| Number of Background Sequences with motif | 12366.5 |
| Percentage of Background Sequences with motif | 37.97% |
| Average Position of motif in Targets | 316.5 +/- 244.0bp |
| Average Position of motif in Background | 312.3 +/- 246.5bp |
| Strand Bias (log2 ratio + to - strand density) | 0.0 |
| Multiplicity (# of sites on avg that occur together) | 1.52 |
| Motif File: | file (matrix) reverse opposite |

### Similar de novo motifs found

|  |  |  |  |  |  |  |  |
| --- | --- | --- | --- | --- | --- | --- | --- |
| Rank | Match Score | Redundant Motif | P-value | log P-value | % of Targets | % of Background | Motif file |
| 1 | 0.961 | G C A T A C T G A T G C A T G C C T G A C T G A C T A G C G T A | 1e-176 | -405.812077 | 60.07% | 45.40% | motif file (matrix) |
| 2 | 0.871 | G C A T G A T C A G C T C A G T C T A G A T C G G T A C G C T A C T G A C T A G T C G A C G T A | 1e-116 | -269.300889 | 38.99% | 27.91% | motif file (matrix) |
| 3 | 0.662 | C T A G T C A G T C G A C G T A G C A T G C A T C T A G A C T G T A G C G C T A | 1e-66 | -153.733647 | 31.61% | 23.71% | motif file (matrix) |
| 4 | 0.677 | C G A T A G T C A G T C C G T A C G T A G A C T C G T A G A T C | 1e-64 | -147.697339 | 36.75% | 28.60% | motif file (matrix) |
| 5 | 0.754 | C T G A T C A G G C T A C G A T G C A T G A T C G A C T C G A T C A T G C A T G G T C A C G T A | 1e-62 | -143.825686 | 14.93% | 9.46% | motif file (matrix) |
| 6 | 0.730 | A G C T G A T C G A C T C A G T C T A G A T C G G T A C G C A T A T G C A C T G | 1e-61 | -141.260644 | 49.37% | 40.84% | motif file (matrix) |
| 7 | 0.736 | A T G C T C A G G C A T C A G T T C A G C A T G G T A C C A G T | 1e-57 | -133.019457 | 39.01% | 31.13% | motif file (matrix) |
| 8 | 0.614 | G A T C C A T G G T A C A C G T C T A G C T A G G T A C C T G A | 1e-46 | -107.338608 | 25.25% | 19.16% | motif file (matrix) |
| 9 | 0.688 | C G A T C T A G T C G A C G T A A T C G G A C T C G A T C A T G T C A G A T G C G C T A T A G C | 1e-44 | -102.970693 | 23.53% | 17.73% | motif file (matrix) |
| 10 | 0.630 | A T G C A G T C C G T A C G T A C G T A A C G T A G T C A C G T A C G T A T C G | 1e-33 | -76.398901 | 63.70% | 57.55% | motif file (matrix) |
| 11 | 0.605 | A C T G A G T C A G T C C G T A C G T A C G A T C T A G A G T C A G T C C G T A C G T A C G T A | 1e-32 | -75.519464 | 49.53% | 43.32% | motif file (matrix) |
| 12 | 0.626 | A G T C G T A C C G T A G T C A C G A T A G T C A C G T A C G T A C G T A C T G A G T C C A T G | 1e-7 | -17.146274 | 0.14% | 0.02% | motif file (matrix) |
